# Associations between the Level of Trace Elements and Minerals and Folate in Maternal Serum and Amniotic Fluid and Congenital Abnormalities

**DOI:** 10.3390/nu11020328

**Published:** 2019-02-03

**Authors:** Rafal Kocylowski, Mariusz Grzesiak, Zuzanna Gaj, Wiktor Lorenc, Ewa Bakinowska, Danuta Barałkiewicz, Constantin S. von Kaisenberg, Yvonne Lamers, Joanna Suliburska

**Affiliations:** 1 Department of Obstetrics, Perinatology and Gynecology, Polish Mother’s Memorial Hospital Research Institute, ul. Rzgowska 281/289, 93-338 Lodz, Poland; rkocylow@gmail.com (R.K.); mariusz.grzesiak@iczmp.edu.pl (M.G.); zuzanna.gaj@iczmp.edu.pl (Z.G.); 2 PreMediCare New Med Medical Center, ul. Drużbickiego 13, 61-693 Poznan, Poland; 3 Scientific Laboratory of the Center of Medical Laboratory Diagnostics and Screening, Polish Mother’s Memorial Hospital Research Institute, ul. Rzgowska 281/289, 93-338 Lodz, Poland; 4 Department of Trace Element Analysis by Spectroscopy Method, Faculty of Chemistry, Adam Mickiewicz University in Poznan, ul. Umultowska 89b, 61-614 Poznan, Poland; wlorenc@amu.edu.pl (W.L.); danutaba@amu.edu.pl (D.B.); 5 Institute of Mathematics, Poznan University of Technology, ul. Piotrowo 3A, 60-965 Poznan, Poland; ewa.bakinowska@put.poznan.pl; 6 Department of Obstetrics and Gynecology, Hannover Medical School, Carl-Neuberg-Str. 1, D-30625 Hannover, Germany; vonKaisenberg.Constantin@mh-hannover.de; 7 Food, Nutrition and Health Program, Faculty of Land and Food Systems, The University of British Columbia, Vancouver, BC V6T 1Z4, Canada; yvonne.lamers@ubc.ca; 8 British Columbia Children’s Hospital Research Institute, Vancouver, BC V5Z 4H4, Canada; 9 Institute of Human Nutrition and Dietetics, Poznan University of Life Sciences, Poznan, Poland, ul. Wojska Polskiego 31, 60-624 Poznan, Poland

**Keywords:** congenital abnormalities, trace elements, minerals, serum, amniotic fluid

## Abstract

Congenital birth defects may result in a critical condition affecting the baby, including severe fetal/neonatal handicap and mortality. Several studies have shown that genetic, nutritional, and environmental factors may have an impact on fetal development and neonatal health. The relevance of essential and toxic elements on fetal development has not yet been fully investigated, and the results of recent research indicate that these elements may be crucial in the assessment of the risk of malformations in neonates. We determined the association between essential and toxic elements and the level of folate in maternal serum (MS) and amniotic fluid (AF), along with neonatal abnormalities. A total of 258 pregnant Polish women in the age group of 17–42 years participated in this study. AF and MS were collected during vaginal delivery or during cesarean section. An inductively coupled plasma mass spectrometry technique was used to determine the levels of various elements in AF and MS. The results of this exploratory study indicate that the levels of essential and toxic elements are associated with fetal and newborn anatomical abnormalities and growth disorders.

## 1. Introduction

The frequency of congenital abnormalities in Poland is approximately 2–4% of live births [1]. They are the primary cause of the deaths in newborns and are the most common cause of physical and intellectual disabilities in children. Cardiac defects were the most frequently identified congenital anomalies in children. In addition to congenital defects, there are many other conditions such as macrosomia, hypotrophy, and systemic disorders that affect neonatal health. Genetic, nutritional, and environmental factors may influence fetal development and neonatal health. However, the etiology of most of the fetal and neonatal abnormalities remains largely unknown [2].

Several studies have shown that micronutrient status plays an important role in the health and well-being of pregnant women and in the development and long-term health of their offspring [3,4]. In a previous study, we found a significant association between the levels of magnesium and copper in AF and fetal biometric parameters [5]. Magnesium levels have been extensively studied in pregnancy, especially due to their role in the prevention and treatment of preeclampsia [6]. A significant association between the intake of magnesium and its levels in pregnancy and birth outcomes has been reported in previous studies [7,8]. A recent study suggests that both maternal deficiency and overload of copper has an adverse effect on neonatal birth outcomes, which may result in preterm birth or late- or post-term birth [9].

Environmental pollutants including heavy metals may also play a contributory role in reproductive and developmental abnormalities [10]. Epidemiological studies indicate an association between exposure to vanadium and low birth weight [11]. Insufficient manganese during pregnancy has been associated with reduced fetal growth [12]. Another study suggests that elevated levels of cadmium in maternal circulation increases the risk of preeclampsia and contributes to the restriction of fetal growth in patients with preeclampsia [13]. The relationship between folate status and both neural tube and cardiac defects has been known for quite some time [14,15,16].

The impact of essential and toxic elements on fetal development has not yet been fully investigated and the mechanisms are poorly understood. The results of recent research indicate that the role of some micronutrients may be crucial in assessing the risk of malformations in infants. Therefore, the aim of this exploratory analysis was to determine the association between essential, toxic elements, and folate; and fetal congenital anatomical defects, fetal growth abnormalities, as well as neonatal disorders.

## 2. Materials and Methods

### 2.1. Study Design

This was an exploratory study to determine the levels of elements and folate in AF and MS between 27 and 42 weeks of gestation. The AF and maternal blood were collected during routine diagnostic and treatment procedures in pregnant women hospitalized in the Polish Mother’s Memorial Hospital Research Institute. The samples were stored at −80 °C.

The study was conducted on pregnant Polish women (white/Caucasian) who were under routine obstetric care. Women voluntarily participated in the study after signing an informed consent form. The participants consented to the secondary use of their samples. The study was conducted on patients qualified to participate based on the inclusion and exclusion criteria.

The inclusion criteria were normal single intrauterine pregnancy and signing an informed consent form. The exclusion criteria were vaginal bleeding, pelvic pain, chorionic hematoma, premature rupture of membranes, placental insufficiency, multiple pregnancies, and genetic conditions in the current pregnancy or present in the women.

The study protocol was approved by the Bioethics Commission of the Research Ethical Committee, Polish Mother’s Memorial Hospital Research Institute in Poland (approval no. 50/2016) and The University of British Columbia Clinical Research Ethics Board (UBC CREB, H16–00419). This study was conducted in accordance with the Declaration of Helsinki. The study has been registered in ClinicalTrials.gov, ID: NCT03598361.

A routine medical interview was conducted to collect data on maternity, pregnancy, family history of illnesses and operations, drugs and medications used, and the occurrence of allergies. Maternal weight, to the nearest 0.1 kg, was measured prior to the delivery using a calibrated digital weight scale while women were wearing lightweight clothing. Maternal height was measured in centimeters and pre-pregnancy weight was self-reported. Pre-pregnancy body mass index was calculated. Birth weight of newborns was assessed using a digital weight scale at the third stage of delivery. The week of gestation was determined by first trimester obstetric ultrasonography.

Congenital birth defects were diagnosed prenatally by performing an ultrasound scan to detect anomalies at 20–24 weeks of gestation according to the guidelines of the Polish Society of Gynecologists and Obstetricians, which are based on the protocols of the International Society of Ultrasound in Obstetrics and Gynecology and Fetal Medicine Foundation. Abnormal prenatal findings and suspected congenital defects were re-evaluated in the neonatal period by pediatricians performing clinical examinations and using ultrasound (for brain scan, ECHO, abdominal scan) and X-ray (chest and lungs) techniques.

Fetal growth disorders, such as macrosomia and fetal growth restriction (FGR)/hypothropy, were established when ultrasound-estimated fetal weight according to the Hadlock formula exceeded the 90th or 10th percentile for gestation, respectively [17]. Gestational age was confirmed at the time of the first trimester scan at 11–14 weeks of gestation by CRL measurement [18]. Abnormal fetal growth patterns were re-evaluated using neonatal weight and growth charts by Fenton et al. [19]. Fetal birth-weight estimation was used only as a screening tool to select growth-affected pregnancies. Finally, the diagnosis of abnormal birth weight was reassessed after delivery with newborn growth charts and neonatal examination as a more accurate method.

Neonatal disorders found during the pediatric postpartum medical examination were classified as neurologic, respiratory, or cardiovascular according to standard newborn assessment protocols [20].

### 2.2. Biospecimen Collection

Blood samples were collected from the antecubital vein of the pregnant women in the first stage of labor (latent phase) during admission to the delivery ward. Monovette test tubes (Neutral or Serum Z/7.5 mL; Sarstedt, Sarstedt AG & Co, Nümbrecht, Germany) were used without any anticoagulants to obtain blood serum and the tubes were spun for 30 min (3000 rpm/min at 4 °C) and then frozen. All serum samples were stored at −80 °C.

Samples of AF were obtained (5 mL) via transabdominal puncture at the surgical wound during cesarean section or transvaginal puncture using a speculum when cervical dilatation was above 4 cm during vaginal birth. The membranes were intact in both situations; sterile needles and syringes were used just before delivery. The samples were centrifuged (3000 rpm/min for 10 min at 4 °C), frozen, and stored at −80 °C.

### 2.3. Folate and Element Measurements

Folate analysis in AF and MS was performed according to the method described in our previous article [21]. We used chloramphenicol-resistant *Lactobacillus casei* as the test microorganism. The inter-assay variations for serum folate concentrations was 5.9%. Whole blood hemolysate (NIBSC code: 95/528) served as an external quality control; the measured mean folate concentration was 12.8 ng/mL (target value 13 ng/mL) with an inter-assay variation of 7.8%. Measurement of essential and toxic elements in AF and MS has been described by Markiewicz et al. [22] and Kocylowski et al. [23]. Samples were mineralized in a high-pressure, closed, microwave digestion system (Ethos One, Milestone). An Elan DRC II ICP-MS (PerkinElmer SCIEX, Ontario, Canada) was used to determine: Mg, Co, Cu, Zn, Sr, Cd, Ba, Pb, U, Ca, Cr, Al, Mn, V, and Fe in AF and MS. Calibration based on a weighted least squares calibration curve was employed for all elements. The linearity—calculated as *R*^2^—was acceptable for all analyzed elements (*R*^2^ > 0.999). The trueness of the analytical method was assessed by analyzing the certified reference material (CRM) Seronorm™Trace Elements Serum L-2. The values of recovery are within an acceptable range for all analytes, which demonstrates that the described analytical procedure is fit for the intended purpose.

### 2.4. Statistical Analysis

**Figure 1:**
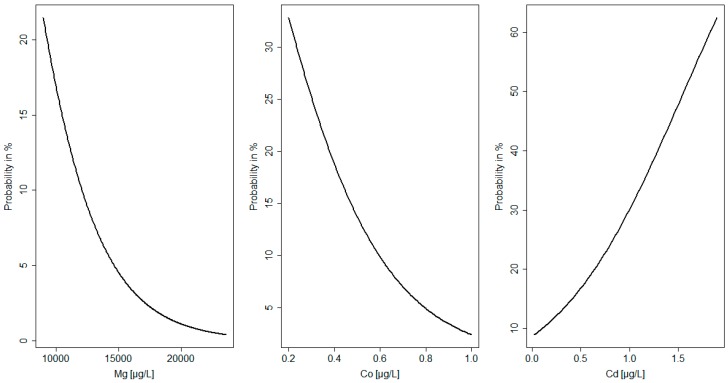
Probability of congenital abnormalities related to element levels in maternal serum.

**Figure 2:**
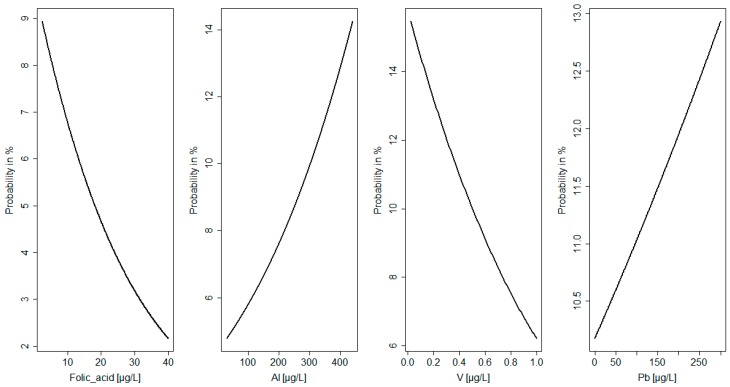
Probability of congenital abnormalities related to element and folate levels in amniotic fluid.

All statistical analyses were performed with Statistica 10 for Windows and RStudio software (R version 3.4.0, R Core Team, Vienna, Austria, 2017). Data were tested for normal distribution using the Shapiro–Wilk test. The Mann–Whitney test was used to compare the differences between groups with defects and a healthy group for all the studied parameters. Logistic regression analysis was used to assess the relationships between variables [24]. The probability function of the anomalies in neonates (e.g., birth defects, macrosomia, and newborn disorders) depending on the values of the elements and folate in the AF and MS was analyzed (Figure 1 and Figure 2). The level of statistical significance was set to *p* < 0.05.

## 3. Results

Table 1 presents the demographic characteristics of the participants. The study population consisted of a total of 258 examined deliveries, of which 56% of the newborns were female and 44% were male. More than 70% of the women gave a normal vaginal birth, while nearly 30% delivered by cesarean section. The median maternal age was 29 years (range, 18–43 years). About 18% of the women were previously smoking and 5% continued smoking during pregnancy. Over 80% of the women had secondary and higher education and 75% declared their socioeconomic status as good or very good.

In this study, we observed a neonatal abnormality rate of nearly 30% in the study population, including congenital birth defects (5.4%), macrosomia (8.1%), hypotrophy (10.9%), and newborn disorders (8.9%). In five cases, macrosomia or birth defects co-occurred with newborn disorders. Nearly 50% of the birth defects were cardiac-related, 30% skeletal defects, and 20% were other disorders (e.g., teratomas). Newborn disorders included cardiovascular, neurological, and respiratory dysfunctions.

The levels of study elements and folate in AF and MS in groups with abnormalities were compared with healthy deliveries as previously published [21,23]. The control group consisted of healthy women without birth defects or any chronic diseases such as diabetes and hypertension, not smoking, without alcohol abuse and preterm birth. The median age of the women, and the socioeconomic status and education in both groups were comparable (Table 2, Table 3 and Table 4). In MS, a significantly lower amount of Mg was found in the birth defects group (I) than that of normal subjects. Co was markedly lower in the group with macrosomia (II) than that of normal subjects. Newborn disorders (IV) were associated with a lower level of folate in the MS, and in the birth defects group (I) the level of folate was non-significantly reduced (*p* = 0.08). Comparison of levels of different trace elements in AF showed the greatest differences in the newborn disorders group (IV), where Al and Ba levels were higher than normal.

The probability function of the anomalies in neonates (e.g., birth defects, macrosomia, and newborn disorders) depending on the values of the elements and folate in the AF and MS was analyzed (Figure 1 and Figure 2). It was found that the probability of birth defects decreased with an increase in Mg concentration in MS. An Mg level below 10,000 µgL^−1^ was related with a 20% probability of the occurrence of birth defects. In maternal serum the probability of macrosomia increased with a decrease in cobalt concentration. A cobalt level below 0.5 µgL^−1^ was related with a nearly 15% probability of the occurrence of birth defects. Moreover, the probability of newborn disorders increased with an increase in cadmium concentration in MS. A cadmium level over 0.5 µgL^−1^ was related with a nearly 20% probability of the occurrence of newborn disorders (Figure 1).

In AF the probability of birth defects decreased with an increase in folate concentration. A folate level below 10 µgL^−1^ was related with a nearly 10% probability of the occurrence of birth defects. It was found that the probability of birth defects increased with an increase in aluminum concentration in AF. In this study, an aluminum level over 300 µgL^−1^ was related with an over 10% probability of the occurrence of birth defects. The probability of macrosomia increased with a decrease in vanadium concentration in AF. In the study group a vanadium level below 0.2 µgL^−1^ was related with an over 10% probability of the occurrence of birth defects. Moreover, it was shown that the probability of newborn disorders increased with an increase in lead concentration in AF. A lead level over 50 µgL^−1^ was related with an over 10% probability of the occurrence of newborn disorders (Figure 2).

The results of the logistic regression analysis showed that, among the selected factors, a high level of Al (β coefficient = −0.28; *p* = 0.02) and low level of folate in AF (β coefficient = 0.24; *p* = 0.01) were the best predictors of birth defects (I). In MS, a low concentration of Mg was related to an increased risk of birth defects (I) (β coefficient = 0.31; *p* = 0.007). Low levels of V in AF (β coefficient = 0.27; *p* = 0.03) and low levels of Co in MS (β coefficient = 0.26; *p* = 0.01) were associated with macrosomia (II). Moreover, high levels of Pb in AF (β coefficient = −0.6; *p* = 0.01) and Cd in MS (β coefficient = −0.15; *p* = 0.03) were predictors of newborn disorders (IV). Figure 1 and Figure 2 show the probabilities of neonatal abnormalities with changing levels of elements and folate in AF and MS.

## 4. Discussion

In this study, we determined the association between trace elements and folate and fetal congenital anatomical defects, fetal growth abnormalities as well as neonatal disorders in a white/Caucasian population. According to the results, low concentrations of Mg, V, Co, and folate in MS and AF in addition to the exposure of pregnant women to Al, Pb, and Cd were associated with an increased risk of fetal and neonatal defects. To the best of our knowledge, this is the first study of Polish pregnant women to demonstrate this relationship.

Our results also showed that the probability of birth defects (over 50% related to cardiac defects) increased with low levels of Mg in MS. Mg is known to play a crucial role in maternal health and fetal development, which is confirmed by the results of this study. This essential mineral is necessary for the growth of the body and plays an important role in the metabolism of the fetus. Its deficiency in pregnancy may increase the risk of health disorders for both the mother and the newborn, such as mortality and morbidity, gestational diabetes, preterm labor, preeclampsia, and small for gestational age or intrauterine growth restriction [21,25]. It is suggested that the influence of Mg on early development relates to the TRPM7 and TRPM6 ion channels that have been implicated in the control of the homeostasis of Mg ions. Gastrulation defects caused by the dysfunction of TRPM7 can be prevented by the supplementation of Mg2+, indicating its essential role in gastrulation and neural fold closure [26]. An experimental study reported placental abnormalities in a mouse model of maternal deficiency of Mg. Placental defects may lead to poor placental perfusion, reduction in litter size, embryonic and neonatal loss, and growth restriction [7]. In mice, maternal hypomagnesemia did not change the activity of TRPM7 and TRPM6 ion channels but increased the expression of placental MagT1 transporter and Ca-selective channels (TRPV6), which probably increases the levels of Mg in the placenta and maintains its function in Mg deficiency [7]. It is not clear if this mechanism is also active during hypomagnesemia in pregnant women. Studies on *Xenopus laevis* have shown that Mg deficiency during embryogenesis inhibits the growth and development of head and heart [26]. Another experimental study indicates that chronic deficiency of Mg decreases in-cardiac tolerance for hypoxia [27]. It seems that a more moderate deficiency of Mg impacts on postnatal growth restriction [7]. Another study has shown that heart failure is related to hypomagnesemia in later life [25].

We found that a high level of Al in AF is related to newborn disorders and that the probability of birth defects increases with increasing levels of Al in AF. This suggests that maternal exposure to Al increases the risk of birth defects and other abnormalities. Al is a toxic metal which easily accumulates in the organism due to its fast intestinal absorption, organic distribution, and rather low excretion. It is transported across the placenta and then to the fetus, and the heart is a potential target of Al toxicity [28]. Results of some experimental studies show that maternal exposure to Al contributes to its bioaccumulation in the heart, which can induce clinical subtypes of congenital heart defects and other cardiac degeneration in offspring. This is potentially mediated by pro-inflammatory and pro-oxidant activities [28,29]. In a recent study conducted with human participants, it has been found that high maternal levels of Al may significantly increase the risk of delivering a child with congenital heart defects or the child may grow up to experience cardiovascular diseases later in life [28,29].

The results of this study indicate that high levels of Pb and Cd in AF and MS are associated with newborn disorders (e.g., neurological, cardiovascular, and respiratory systems). Results from human studies have shown an association between As, Cd, Mn, and Pb pollution and their high levels in MS and congenital heart defects in the fetus [10,30,31]. Elevated levels of Pb in umbilical serum is related to oxidative stress and the occurrence of cardiovascular diseases in newborns [30]. Experimental studies have supported the evidence that Cd might impair placental blood circulation, inhibiting the transport of nutrients from the mother to the fetus [32]. Maternal exposure to Cd during organogenesis in mice disturbs transport of folate from the maternal circulation to the fetuses through the downregulation of placental folate transporters and may induce neural tube defects [33]. In turn, Wang et al. [34] suggest that maternal exposure to Cd during pregnancy decreases placental transport of Zn and induces fetal growth restriction in pregnant mice. In human studies, high levels of Cd in maternal urine is associated with low birth weight [35]. However, in this study, we did not find a connection between heavy metals and hypotrophy. The results obtained in experiments with rats suggested that adequate intake of Zn may protect against Cd toxicity [36]. Some authors suggest that the toxic effects of heavy metals on the organism depend on the variations in the metabolism of heavy metals across different populations [35].

In this study, we found that V and Co are the important elements needed for fetal growth, and a low level of these elements is related to a high probability of macrosomia. V is an essential element and is necessary for the normal growth and development of bone and reproductive systems [37]. To our knowledge, this study is the first to suggest the association between low levels of V in AF and macrosomia. Other studies have confirmed a negative effect of prenatal exposure to V on preterm delivery, restricted birth weight, and increased risk of adverse birth outcomes [11,37,38]. However, we measured V in MS and AF and in several other studies V was assessed in the urine. The results obtained in this study may be related to the fact that macrosomia is strongly associated with pre-pregnancy overweight and obesity, and in obese people, low concentrations of V and Co have been reported [39,40,41]. Therefore, obese women with low levels of V and Co may be at high risk of fetal macrosomia. In a recent human study, it has been found that the level of Co in the placenta is a significant predictor of birth weight, which was demonstrated as a non-linear relationship between Co levels in the placenta with birth weight [42].

The results of our study have confirmed that low maternal folate status during pregnancy is associated with a high risk of birth defects. Several studies have indicated that folate deficiency during embryogenesis in pregnancy is related to neural tube defects [16,43,44]. In addition, the risk of conotruncal cardiac defects increases with genetic disturbances in folate metabolism [16]. In congenital heart defects, elevated levels of homocysteine have been observed in neonates, which may be the result of disturbances in the folate metabolism in their mother [45]. However, a previous study did not demonstrate any significant relationship between MS folate status in the first trimester and birth defects [46].

The limitations of the study are: firstly, analysis of elements and folate only in serum and AF, not umbilical cord blood, which may better clarify the impact of essential and toxic elements and folate on the fetuses. Secondly, in this study, we included only women at delivery and a rather heterogeneous group of birth defects and fetal growth abnormalities. Furthermore, in the present study, we did not determine nutritional factors and environmental factors that may affect the concentrations of elements in the analyzed samples. Another limitation involves the maternal and obstetrical backgrounds which differ between the present and previous studies. The former study was designed to create reference ranges based only on healthy non-smoking women with normal growth pregnancies. The latter research also included smoking patients, women with chronic diseases, and pregnancy-related conditions. These are potential confounding factors which may affect essential and toxic elements and folate, and thus should be considered in the interpretation of the results and in future study designs.

## 5. Conclusions

In conclusion, low concentrations of Mg and folate in the MS and AF increase the risk of birth defects. Macrosomia is related to low levels of V in AF and Co in MS. Maternal exposure to Al is associated with birth defects, especially cardiac dysfunction. Furthermore, high levels of Pb and Cd in AF and MS increase the risk for neurological, cardiovascular, respiratory, and other disorders in newborns after birth. The results of this study indicate that essential and toxic elements are associated with fetal congenital anatomical defects and growth disorders. Our results provide directions for further research.

## Figures and Tables

**Table 1:** Characteristics of the total population (mean ± SD/median/min-max).

Maternal Parameters		Newborn Parameters	
Parameter	Value	Parameter	Value
Number of women	258	Gender	
		F/M	144/114
Age of mother (y)	29.4 ± 4.8	Birth weight (g)	3228.5 ± 595.2
	29		3300
	18–43		1200–5000
Weight of mother in pregnancy (kg)	77.4 ± 12.8 76 46–124	Vaginal delivery (%) Cesarean section (%)	72% 28%
BMI of mother before pregnancy (kg/m^2^)	22.6 ± 3.9	Birth defects (%)	5.4
	21.7		
	16.3–40.8		
Week of gestation	38.3 ± 2.2	Macrosomia (%)	8.1
	39		
	27–42		
Gravidity/Parity (%)		Hypotrophy (%)	10.9
0	51/57		
1	31/35		
2	12/6		
3	3/1		
≥4	3/1		
Smoking		Newborn disorders (%)	8.9
Before pregnancy (%)	18.2		
During pregnancy (%)	5		
Disorders during pregnancy (%)			
Hypertension	10		
Diabetes	5		
Inflammation	4		
Education (%)			
Primary school	2		
Vocational school	5		
High school	25		
University	68		
Socioeconomic status (%)			
Poor	4		
Moderate	21		
Good	69		
Very good	6		

Note: SD, standard deviation; min, minimum; max, maximum; y, years; F, female; M, male.

**Table 2:** Trace elements in maternal serum; mean ± SD/median/min-max (µg L^−1^).

	Birth Defects	Macrosomia	Hypotrophy	Newborn Disorders
Mg	13,274 ± 3482 *	14,838 ± 2797	15,471 ± 2999	14,519 ± 2309
Co	0.55 ± 0.19	0.52 ± 0.13 *	0.61 ± 0.18	0.59 ± 0.14
Cu	2161.0 ± 389.0	2158.1 ± 458.1	2077 ± 445.7	2271.7 ± 362.3
Zn	1056.9 ± 408.4	913.9 ± 194.2	953.8 ± 351.8	950.4 ± 359.4
Sr	53.0 ± 21.7	124.4 ± 117.9	126.1 ± 125.3	114.1 ± 132.4
Cd	0.18 ± 0.12	0.13 ± 0.08	0.13 ± 0.10	0.17 ± 0.08
Ba	17.61 ± 11.54	17.53 ± 11.4	18.73 ± 11.06	14.64 ± 11.03
Pb	1.56 ± 0.80	5.95 ± 10.98	6.98 ± 12.86	2.63 ± 6.99
U	0.32 ± 0.13	0.32 ± 0.07	0.35 ± 0.12	0.32 ± 0.12
Ca	96,142 ± 7491	97,220 ± 20,009	99,184 ± 21,951	92,597 ± 15,830
Cr	4.61 ± 4.07	5.49 ± 5.86	5.10 ± 4.39	3.28 ± 2.95
Al.	250.3 ± 176.2	283.0 ± 176.1	369.8 ± 216.6	300.6 ± 152
Mn	14.1 ± 58.12	15.41 ± 18.64	10.30 ± 4.86	13.05 ± 7.20
V	0.27 ± 0.16	0.28 ± 0.1	0.33 ± 0.14	0.26 ± 0.11
Fe	1475 ± 569.8	1257.7 ± 496.4	1218.1 ± 581.8	1136.2 ± 507.5

Note: Min, minimum value; max, maximum value; SD, standard deviation. * *p* < 0.05 significant differences vs. healthy women [23].

**Table 3:** Trace elements concentration in amniotic fluid; mean ± SD/median/min-max (µg L^−1^).

	Birth Defects	Macrosomia	Hypotrophy	Newborn Disorders
Mg	8363 ± 2794	9511 ± 2462	9948 ± 2959	10,107 ± 2521
Co	0.24 ± 0.05	0.26 ± 0.08	0.27 ± 0.11	0.27 ± 0.09
Cu	79.87 ± 23.66	75.70 ± 25.69	78.44 ± 27.69	82.31 ± 23.66
Zn	400.8 ± 218.7	579.8 ± 328.4	427.6 ± 193.6	454.2 ± 260.7
Sr	67.82 ± 62.34	75.82 ± 50.88	75.83 ± 65.22	72.87 ± 62.85
Cd	0.09 ± 0.03	0.13 ± 0.11	0.12 ± 0.09	0.12 ± 0.10
Ba	12.03 ± 4.65	12.40 ± 0.42	12.90 ± 10.52	14.08 ± 8.02 *
Pb	44.20 ± 66.11	36.28 ± 49.34	49.60 ± 67.82	54.96 ± 101.99
U	0.02 ± 0.01	0.06 ± 0.12	0.05 ± 0.06	0.04 ± 0.06
Ca	61,084 ± 19,355	73,397 ± 23,957	76,052 ± 22,226	69,302 ± 16,239
Cr	3.18 ± 2.89	2.61 ± 2.38	3.05 ± 3.05	3.29 ± 3.36
Al.	144.8 ± 54.1	133.1 ± 65.2	140.8 ± 58.38	165.77 ± 91.17 *
Mn	4.41 ± 1.28	6.20 ± 3.30	5.89 ± 2.98	5.66 ± 2.93
V	0.26 ± 0.13	0.27 ± 0.19	0.33 ± 0.20	0.31 ± 0.18
Fe	470.3 ± 298.8	491.1 ± 191.1	479.3 ± 255.0	502.8 ± 278.1

**Note:** Min, minimum value; max, maximum value; SD, standard deviation. * *p* < 0.05 significant differences versus healthy women [23].

**Table 4:** Folate concentration; mean ± SD/median/min−max (nmol).

	Birth Defects	Macrosomia	Hypotrophy	Newborn Disorders
AF	10.93 ± 7.15	11.51 ± 7.32	10.91 ± 9.42	10.93 ± 8.12
MS	41.63 ± 23.03	46.33 ± 22.28	47.20 ± 28.52	40.33 ± 24.80 *
UCB	83.62 ± 34.89	84.95 ± 35.85	74.99 ± 24.37	79.51 ± 37.70

**Note:** Min, minimum value; max, maximum value; SD, standard deviation. * *p* = 0.04 significant differences vs. healthy women [21].
